# Modeling and forecasting carbon dioxide emission in Pakistan using a hybrid combination of regression and time series models

**DOI:** 10.1016/j.heliyon.2024.e33148

**Published:** 2024-06-19

**Authors:** Hasnain Iftikhar, Murad Khan, Justyna Żywiołek, Mehak Khan, Javier Linkolk López-Gonzales

**Affiliations:** a Department of Statistics, Quaid-i-Azam University, Islamabad, 45320, Pakistan; b Department of Statistics, Abdul Wali Khan University Mardan, Mardan, 23200, Pakistan; c Faculty of Management, Czestochowa University of Technology, Czestochowa, 42-200, Poland; d Department of Computer Science, Electrical Engineering and Mathematical Sciences, Western Norway University of Applied Sciences, Bergen, 5063, Norway; e Escuela de Posgrado, Universidad Peruana Unión, Lima, 15468, Peru

**Keywords:** Carbon emission forecasting, Regression methods, Time series methods, Hybrid combination models, Energy policy

## Abstract

Carbon dioxide (CO_2_) emissions continue to rise globally despite efforts to combat climate change. Energy industry emissions are a pressing global issue, causing devastating impacts. Hence, it is vital to accurately and efficiently forecast CO_2_ emissions. Thus, this study comprehensively analyzes forecasting CO_2_ emissions by comparing various hybrid combinations of regression and time series methods to explore the CO_2_ emissions in Pakistan. First, divide the yearly time series of CO_2_ emissions into the long-run curve trend series and the residual subseries. The long-run curve trend subseries is modeled using parametric and nonparametric regression methods, while various standard time series models are used to forecast the residual subseries. However, the forecasts of each subseries will be combined to obtain the final forecast of CO_2_ emissions. This work used four different accuracy mean errors, a statistical test, and a graphical analysis as performance measures to evaluate the proposed hybrid forecasting technique. The findings confirmed that the proposed hybrid combination forecasting technique is highly accurate and efficient in forecasting CO_2_ emissions. Likewise, according to the proposed final optimal hybrid combination forecasting model, Pakistan's per capita CO_2_ emissions will be 1.130215 metric tons in 2030. Pakistan's escalating emission trend signals that creative solutions must be implemented to curb it. Thus, the government must price carbon footprints, regulate electricity from zero-carbon sources, reduce population, encourage afforestation in densely populated areas, adopt clean technology, and fund research.

## Highlights

• The study presents hybrid models to forecast CO_2_ emissions in Pakistan.
• Building hybrid models are based on different regression and time series models.
• These models accurately forecast CO_2_ emissions based on various metrics and tests.
• Study forecasts that Pakistan's CO2 emissions will reach 1.130215 metric tons by 2030.
• Which necessitates innovative environmental measures.

## 1 Introduction

Human activities have significantly increased greenhouse gas (GHG) concentration and emissions since 1750 [1]. GHGs trap heat near the Earth's surface and are crucial for maintaining Earth's habitable temperature [2]. However, excessive consumption of fossil fuels and other resources contributes to GHG overburden, potentially disrupting the Earth's carbon cycle and contributing to global warming. The primary human-emitter activities include electricity generation, heat, and transport, with energy consumption contributing around 73% to emissions. Deforestation, the fossil-fuel industry, livestock, and fertilizer also contribute to 18% of emissions of CO_2_, methane, and nitrous oxide [3], [4], [5]. The global CO_2_ emissions in 2022 were 36.1 ± 0.3 GtCO_2_, reflecting 2%, 7.9%, and 1.5% growth compared to 2019, 2020, and 2021, respectively [6], [7]. The Carbon Monitor data suggested 1.5% (0.9-2.6%) growth for the year 2021 to 2022, along with other energy consumption-based projected growth of 1.0 ± 0.9% by Global Carbon Project (CGP) and the International Energy Agency (IEA) [8], [9], [10].

In 2020, COVID-19 restrictions decreased global CO_2_ emissions, but they have since increased beyond pre-pandemic levels [11], [12]. This suggests that the peak CO_2_ emissions may not have reached yet, and the temperature may hit 5 °C before 2030 pre-industrial levels, the limit set by G7 countries and the Paris Agreement [13], [14], [15]. Developed countries contribute more CO_2_ emissions due to their energy industries and transportation, causing higher carbon emissions. China is the world's largest emitter of CO_2_, emitting around 10 billion tonnes annually, accounting for 28.8% of global emissions [16], [17], [18]. This rapid increase in emissions has led to catastrophes like floods, droughts, rising healthcare issues, and other global warming and climate change calamities [19], [20], [21], [22]. Pakistan, the 19th country with the highest GHG emissions, has been severely affected by devastating floods, affecting 33 million people and causing 1,700 deaths [23], [24], [25]. With a growing population of 220 million, Pakistan is expected to emit 2.9 tonnes per capita of CO_2_ by 2025 and 5.4 tonnes per capita by 2050. To address these issues, stakeholders need to adopt productive and efficient policies. Pakistan's agriculture sector is also affected by population growth, leading to deforestation and increased CO_2_ emissions [26], [27], [28].

The growing population also causes an increase in automobiles, with carbon emissions used in the country. The government of Pakistan is trying to mitigate and reduce CO_2_ emissions by adopting specific programs that include technology-based innovations and nature-based solutions and launching a mass-scale program of planting ten billion trees Tsunami. These were found very beneficial in providing livelihood opportunities to the youth and reducing the emission to some extent from the high-emission industry and energy sector [29], [30]. However, these steps can further be strengthened by proper planning, policy-making, and time decision-making to meet the conditionally targeted goals set in the Paris Agreement to reduce an overall emission of 50% by 2030, with a reduction of 15% from own resources and 35% from the expected grants from international finance. In addition, Pakistan aims to shift 30% on electric vehicles, 60% on renewable energy, and to impose a ban on coal imports along with expanding nature-based solutions by 2030 [31], [32]. Therefore, these challenges motivate us to choose Pakistan for this research, and it aims to assist governments in making policies and achieving their targeted goals in the light of efficient modeling and forecasting of CO_2_ emission in Pakistan.

Statistical, machine learning (ML), time series, and hybrid models are widely used in the literature for modeling and forecasting CO_2_ emissions [33], [34], [35], [36], [37]. Pakistan is one of the top victims of climate change, and it is very crucial to forecast CO_2_ emissions here; therefore, the researchers in [38] used a univariate model, ARIMA (autoregressive integrated moving average) model for CO_2_ emission forecast of 2030 based on the developed scenario of CPEC (China Pakistan Economic Corridor) in which most of the projects are energy oriented. They attempted to estimate emission reduction percentages and assist the government in adopting suitable policies accordingly. Their findings last revealed the forecast with less than 10% mean absolute percentage error. The authors in [39] used the time series models, including the ARIMA model, the Holt-Winters models, and the seasonal ARIMAX model with exogenous factors and ML and deep learning models such as Linear Regression, Random forest, long short-term memory (LSTM) models to predict ten years CO_2_ emission in India. Their results revealed that LSTM, SARIMAX, and Holt-Winters are the best performers of the six used models regarding the highest accuracy and lowest mean errors in predicting CO_2_ emissions. The energy sector, i.e., transport and manufacturing sectors, is a leading factor in CO_2_ emissions in Pakistan. Therefore, the researchers in [40] evaluated the performance of statistical and ML models for forecasting sector-wise CO_2_ emission in Pakistan till 2030 and provided valuable suggestions to the government for making better decisions based on the produced results.

Unlike statistical, ML, and time series models, many researchers have created hybrid models to increase forecasting accuracy and efficiency by combining features from many methods [41], [42], [43], [44], [45]. For example, the researchers in [46] developed a hybrid approach of ETS and ANN based on combining linear and nonlinear exponential smoothening models to obtain various compositions of linear and nonlinear patterns in time series forecasting. It achieved the best results compared to ARIMA, ETS, MLP, and the hybrid ARIMA-ANN model [47]. Further, the researchers in [48] used the Bi-LSTM model to predict CO_2_ emissions in South Asian countries and China from 2022 to 2030. The effects of technological, industrial, and scientific sectors on CO_2_ emissions are studied. The results of Bi-LSTM yield the comparatively lowest mean errors compared to LSTM and Gated Recurrent Unit (GRU). Their finding declared carbon emissions a big issue, and it will become worse if India and China fail to control high emissions in the next decade. In further studies [49] to find the relationship of gross domestic product (GDP) in Bangladesh with CO_2_ emission due to the consumption of electrical energy by using the fully modified ordinary least squares (FMOLS) technique, convolutional neural network (CNN), dense neural network (DNN), LSTM, and CNN-LSTM. These models were comparatively evaluated with performance measures including RMSE, MAE, and MAPE, and the results suggest a significant effect of CO_2_ emissions over GDP. The findings revealed in [50] that the main contributor to CO_2_ emissions is energy demand in the case of Iran, achieved by applying the Logarithmic Mean Divisia Index decomposition technique.

To motivate the above hybrid models, this work proposes various hybrid forecasting models based on parametric and nonparametric regression models with the combination (hybridization) of linear and nonlinear time series models to enhance the forecasting accuracy and efficiency of CO_2_ emissions in Pakistan. The polynomial regression and spline regression models are considered in regression models. However, in the time series models, consider four standard models: two linear models, the autoregressive model, the autoregressive moving average, and two nonlinear models, the nonparametric autoregressive model and the neural network autoregressive model. In contrast, in the literature on hybrid models, the direct hybridization of two or more models was not primarily the per-define components; they only applied the model. To this end, the main difference between the current work and the above literature is that this work discusses the time series database capture by the parametric and nonparametric regression models. The reaming series is used to model different time series models. Obtained the forecast for these regression and time series models and considered them the final estimates. In addition, the proposals can also be used for hourly, daily, weekly, and monthly time series datasets because they are worth handling other seasonality in the database, which the mentioned literature hybrid models did not offer.

Thus, this study thoroughly examines predicting CO_2_ emissions by comparing sixteen distinct hybrid combinations of regression and time series methods to investigate CO_2_ emissions in Pakistan. The polynomial regression and spline regression models are considered in regression models. However, in the time series models, consider four standard models: two linear models, the autoregressive model, the autoregressive moving average, and two nonlinear models, the nonparametric autoregressive model, and the neural network autoregressive model. In this context, the performance of all possible hybrid combinations of the parametric and nonparametric regression models and the considered time series will be compared using the accuracy metrics, a statistical test, and visual analysis within the proposed hybrid combination methodology. In addition, the performance of the proposed hybrid combination forecasting approach compared to the established benchmark models will be evaluated. However, within the proposed hybrid combination technique, the final best hybrid combination model is compared to the unique time series models (the autoregressive, the nonparametric autoregressive, the autoregressive moving average, and the neural network autoregressive), and the outcomes are noted. Thus, the reported results demonstrated that the proposed final best hybrid combination model is very accurate and efficient for projecting CO_2_ emissions compared to all the considered benchmark models. Finally, while this study only employs the CO_2_ emission information from Pakistan, it may be extended and adapted to other countries to ensure the efficacy and accuracy of the proposed hybrid combination modeling and forecasting technique.

The rest of the paper is organized in the following order: Section 2 describes the models used in developing the proposed hybrid combination modeling and forecasting strategy. Section 3 presents a practical implementation of the proposed hybrid combination forecasting technique utilizing Pakistan's annual CO_2_ emissions. Section 4 discusses the best combination model with a comparison to some of the best time series. Also, it discusses the final observations policy advice for stakeholders. Finally, Section 5 addresses the conclusion, limits, and future research endeavors.

## 2 Method and materials

This section thoroughly reviews the models and methods for building the proposed hybrid combination forecasting technique. As a result, turn to the following subsections for further information on each model and approach.

### 2.1 The proposed hybrid modeling and forecasting procedure

This section will discuss the whole procedure of the proposed hybrid combination forecasting technique. Let the ${\text{C}}_{\text{y}}$ represent the time series of CO_2_ for the year y^th^. The graphical presentation of the ${\text{C}}_{\text{y}}$ time series is shown in 3. It can be seen from this figure the ${\text{C}}_{\text{y}}$ time series have an increasing upward trend. To achieve this, divide the yearly time series of CO_2_ emissions into two new series: the long-run curve trend series and the residual series. However, the mathematical equation can be written as;

(1) $${\text{C}}_{\text{y}}={\text{L}}_{\text{y}}+{\text{R}}_{\text{y}}$$

In the equation (1), ${\text{C}}_{\text{y}}$ represents the time series of CO_2_ emissions; ${\text{L}}_{\text{y}}$, a long-run trend curve series, and ${\text{R}}_{\text{y}}$, a residual series, to model the long-run curve trend series (${\text{L}}_{\text{y}}$) using parametric and nonparametric regression methods. Parametric regression includes polynomial regression, whereas nonparametric regression includes the smoothing regression splines method. However, on the other hand, for residual series (${\text{R}}_{\text{y}}$) modeling, we consider four different univariate time series models: two linear time series models, the autoregressive model, and the autoregressive moving average; and two nonlinear models: the nonparametric autoregressive model and the neural network autoregressive model. Hence, the trend and residual series modeling details are in the coming section.

#### 2.1.1 Modeling the long-run trend series

In this section, we will discuss how to forecast the long-run curve trend series using parametric and nonparametric regression methods. To achieve this, we will model the response variable ${\text{C}}_{\text{y}}$ linearly by estimating the long-run trend component ${\text{L}}_{\text{y}}$ using a cubic polynomial regression technique for time y. The mathematical form is given by

(2) $${\text{L}}_{\text{y}}=\phi_{0}{\text{L}}_{\text{y}}+\phi_{1}{\text{L}}_{\text{y}}^{2}+\phi_{2}{\text{L}}_{\text{y}}^{3}$$

The regression coefficients of the above equation are determined using the ordinary least square method; after obtaining $\overset{ˆ}{\phi_{0}},\overset{ˆ}{\phi_{1}},\text{ and }\overset{ˆ}{\phi_{2}}$, the polynomial regression model coefficients, the final estimated equation is presented.

(3) $${\overset{ˆ}{\text{L}}}_{\text{y}}=\overset{ˆ}{\phi_{0}}{\text{L}}_{\text{y}}+\overset{ˆ}{\phi_{1}}{\text{L}}_{\text{y}}^{2}+\overset{ˆ}{\phi_{2}}{\text{L}}_{\text{y}}^{3}$$

The long-run trend series can be modeled nonparametrically in the nonparametric case.

(4) $${\text{L}}_{\text{y}}={\text{f}}_{1}({\text{L}}_{\text{y}})+{\text{f}}_{2}({\text{L}}_{\text{y}})+{\text{f}}_{3}({\text{L}}_{\text{y}})$$

In the equation (4), each ${\text{f}}_{\text{i}}$ is a smoothing function of ${\text{L}}_{\text{y}}$. As a function of time y, the long-term trend component ${\text{L}}_{\text{y}}$ is estimated. Cubic regression splines assess the long-run trend part for smoothing functions. The number of knots and their position are the most critical choices in the regression spline technique since they dictate the smoothness of the approximation. For this problem, we employ the cross-validation approach. However, the ordinary least squares method is used to calculate regression coefficients.

**Figure 1:**
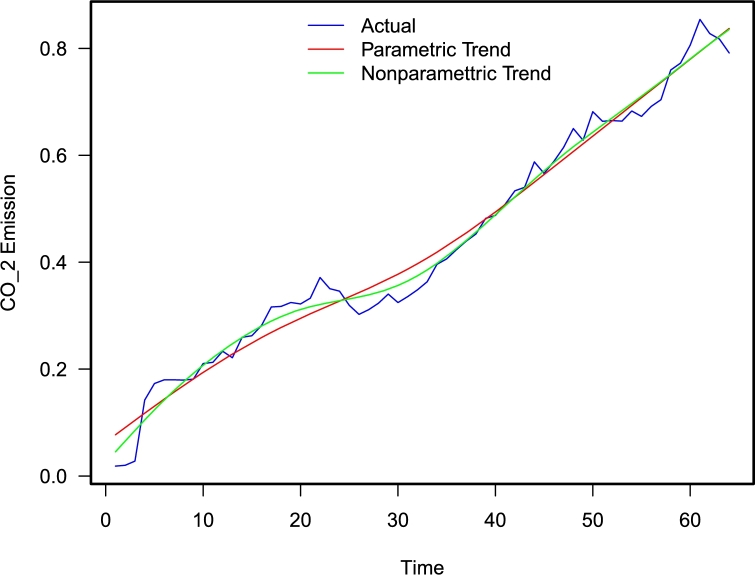
Pakistan CO_2_ emission time series with superimposed fitted long run trend series (L_y_) for polynomial regression model (red curve) and spline regression model (green line).

The observed CO_2_ emission series and the estimated long-run trend curve series are depicted in Fig. 1, with a parametric model (red line) and a nonparametric model (green curve) to visualize the performance of the approaches discussed above used for the estimation of long-run trend curve series ${\text{L}}_{\text{y}}$ (both parametric and nonparametric). As the growing (upward) trend can be observed in the figure, both models employed to estimate ${\text{L}}_{\text{y}}$ adequately capture the long-run trend curve series of CO_2_ emission series (${\text{C}}_{\text{y}}$).

Once the estimated long-run trend curve is obtained, the residual series can be derived as follows:

(5) $${\text{R}}_{\text{y}}={\text{C}}_{\text{y}}-({\overset{ˆ}{\text{L}}}_{\text{y}})$$

#### 2.1.2 Modeling to residual series

After modeling the long-run trend series (${\text{L}}_{\text{y}}$) from the original time series of CO_2_ (${\text{C}}_{\text{y}}$)using the polynomial regression (parametric case) and regression spline (nonparametric case), the next step is to forecast the residual series (${\text{R}}_{\text{y}}$) using four commonly time series models including, the parametric autoregressive model, the autoregressive moving average model, the nonparametric autoregressive model, and the neural network autoregressive model. These models are detailed in the following subsections.

#### 2.1.3 Parametric autoregressive model

A linear and parametric autoregressive (AR) model, which uses a linear combination of the previous observations of the ${\text{R}}_{\text{y}}$ time series and represents the short-term dynamics of this series, is referred to as:

(6) $${\text{R}}_{\text{y}}=\alpha +\vartheta_{1}{\text{R}}_{\text{y-1}}+\vartheta_{2}{\text{R}}_{\text{y-2}}+....+\vartheta_{\text{n}}{\text{R}}_{\text{y}-\text{n}}+\varepsilon_{\text{n}}$$

In the above formula, *α* is an intercept term, and $\vartheta_{\text{j}}(\text{j}=1,2,\cdots ,\text{n})$) is the slope parameter of the underlying AR process, and $\varepsilon_{\text{d}}$ is the disturbance term [51]. However, the present study uses the maximum likelihood method to estimate the parameters of the AR model. The model includes lags 1, 2, and 3 based on their significant results in the series' correlogram (autocorrelation and partial autocorrelation functions).

#### 2.1.4 Autoregressive moving average model

The autoregressive moving average (ARMA) model integrates the target variable's previous values and vital information as moving averages (the error lags). In our scenario, the study variable ${\text{R}}_{\text{y}}$ is explained by the prior terms, as are the delayed residual values [52]. Mathematically,

(7) $${\text{R}}_{\text{y}}=\alpha +\vartheta_{1}{\text{R}}_{\text{y-1}}+\vartheta_{2}{\text{R}}_{\text{y-2}}+\cdots +\vartheta_{n}{\text{R}}_{\text{y}-\text{n}}+\varepsilon_{\text{n}}+\zeta_{1}\varepsilon_{\text{y}-1}+\zeta_{2}\varepsilon_{\text{y}-2}+....+\zeta_{\text{m}}\varepsilon_{\text{y}-\text{m}}$$

In the last equation, *α* denotes intercept, $\vartheta_{\text{j}}(\text{j}=1,2,\cdots ,\text{n})$ and $\zeta_{\text{k}}(\text{k}=1,2,\cdot ,\text{m})$ are the parameters of AR and MA process respectively, and $\varepsilon_{\text{n}}$ is a Gaussian white noise series with mean zero and variance $\sigma_{\varepsilon }^{2}$. Hence, the maximum likelihood method estimates the unknown population parameters. The ARMA model order selection, which is the number of past values and the past error term value, is established by examining the correlograms. In the MA part, the first two lags are significant, while in the AR part, only lags 1, 2, and 3 are significant for the residual time series (${\text{R}}_{\text{y}}$).

#### 2.1.5 Nonparametric autoregressive model

The nonlinear or nonparametric alternative of the AR process leads to the additive model (NPAR), in which the relationship between ${\text{R}}_{\text{y}}$ and its prior terms does not have any specified parametric form, allowing for any nonlinearity, which is stated as:

(8) $${\text{R}}_{\text{y}}={\text{f}}_{1}({\text{R}}_{\text{y-1}})+{\text{f}}_{2}({\text{R}}_{\text{y-2}})+\ldots +{\text{f}}_{\text{y}}({\text{R}}_{\text{y - n}})+\varepsilon_{\text{n}}$$

where ${\text{f}}_{\text{j}}(\text{j}=1,2,\cdots ,\text{n})$ depict smoothing functions and define the relationship between ${\text{R}}_{\text{y}}$ and its past values [53]. As a result, the functions ${\text{f}}_{\text{i}}$ are represented by cubic regression splines in this study and lags 1, lag 2, and lag 3 are employed for NPAR modeling.

#### 2.1.6 Neural network autoregressive model

A neural network autoregressive (NNA) machine learning model forecasts future time series values based on past observations [54]. The model employs a mathematical function that takes into account the past values of the time series, denoted by ${\text{R}}_{\text{y}-1},{\text{R}}_{\text{y}-2},...,{\text{R}}_{\text{y}-\text{n}}$, where n is the time delay parameter. The NNA model is trained using backpropagation and the steepest descent strategy to reduce the discrepancy between anticipated and actual values.

An NNA model forecasting procedure consists of two parts. First, the order of autoregression is calculated, which refers to the number of prior values required to forecast the time series' present value. Second, the NN is trained with a training set that considers the order of autoregression. In univariate time series forecasting, the number of input nodes is determined by the order of autoregression, and the inputs are the prior, lagged data. The anticipated values represent the NN model's output. As there is no theoretical foundation for setting them, trial and error or experimentation frequently establish the number of hidden nodes. It is essential to select the number of iterations carefully to avoid overfitting. In this work, an NNA (3,2) design is used, which can be represented as ${\text{R}}_{\text{y}}=\text{f}({\text{R}}_{\text{y-1}})$. Here, ${\text{R}}_{\text{y-1}}=({\text{R}}_{\text{y-1}},{\text{R}}_{\text{y-2}},{\text{R}}_{\text{y-3}}$) is a vector containing past values of the residual time series of (${\text{R}}_{\text{y}}$), and f is a neural network with 3 hidden nodes in a signal layer.

### 2.2 Accuracy measures

The proposed hybrid combination approach is evaluated using three criteria: a) accuracy mean errors, b) statistical tests, and c) graphical presentation. The considered accuracy mean errors are the following: Mean Absolute Error (MAE), Mean Absolute Percentage Error (MAPE), Root Mean Square Error (RMSE), and Root Mean Square Percentage Error (RMSPE). The functional form of each error is given by;

(9) $$\text{MAE}=\frac{1}{\text{y}}\sum_{\text{y}=1}^{\text{y}}\left(|{\text{C}}_{\text{y}}-{\overset{ˆ}{\text{C}}}_{\text{y}}|\right),$$

(10) $$\text{MAPE}=\frac{1}{\text{y}}\sum_{\text{y}=1}^{\text{y}}\left(\frac{\left|{\text{C}}_{\text{y}}-{\overset{ˆ}{\text{C}}}_{\text{y}}\right|}{\left|{\text{C}}_{\text{y}}\right|}\right)\times 100,$$

(11) $$\text{RMSPE}=\sqrt{\frac{1}{\text{y}}\sum_{\text{y}=1}^{\text{y}}{\left(\frac{\left|{\text{C}}_{\text{y}}-{\overset{ˆ}{\text{C}}}_{\text{y}}\right|}{\left|{\text{C}}_{\text{y}}\right|}\right)}^{2}}\times 100,$$

(12) $$\text{RMSE}=\sqrt{\frac{1}{\text{y}}\sum_{\text{y}=1}^{\text{y}}{\left({\text{C}}_{\text{y}}-{\overset{ˆ}{\text{C}}}_{\text{y}}\right)}^{2}},$$

where ${\text{C}}_{\text{y}}$ is the actual value and ${\overset{ˆ}{\text{C}}}_{\text{y}}$ is the projected value for the ${\text{y}}^{\text{th}}$ value (y =1, 2, …, Y = 20). However, The smaller the MAE, MAPE, RMSPE, and RMSE values, the better the model's forecasting accuracy.

In contrast to the performance indicators described above, the Diebold-Mariano (DM) [55] test is a frequently employed statistical test in the literature for assessing forecasts from different forecasting models [56], [57], [58], [59]. For example, consider the two forecasts produced by two distinct time series models that include as ${\overset{ˆ}{\text{C}}}_{1\text{y}}$ (forecasted values of model 1) and ${\overset{ˆ}{\text{C}}}_{2\text{y}}$ (forecasted values of model 2). However, ${\text{Q}}_{1\text{d}}={\text{C}}_{\text{y}}-{\overset{ˆ}{\text{C}}}_{1\text{y}}$ and ${\text{Q}}_{2\text{y}}={\text{C}}_{\text{y}}-{\overset{ˆ}{\text{C}}}_{2\text{y}}$ are the respectively forecast errors. The loss associated with forecast error ${\left\{{\text{Q}}_{\text{i}\text{y}}\right\}}_{\text{i}=1}^{2}$ by $\text{L}({\text{Q}}_{\text{i}\text{y}})$. For instance, time y absolute loss would be $\text{L}({\text{Q}}_{\text{i}\text{y}})=|{\text{Q}}_{\text{i}\text{y}}|$. For time y, the loss resulting from forecasts 1 and 2 is so ${\text{z}}_{\text{y}}=\text{L}({\text{Q}}_{1\text{y}})-\text{L}({\text{Q}}_{2\text{y}})$. The null hypothesis with the same accuracy in forecasting for two forecasts is $\text{E}[{\text{z}}_{\text{y}}]=0$. The DM test requires that the loss difference have a constant covariance, that is,

(13) $$\text{E}[{\text{z}}_{\text{y}}]=\text{u},\;\forall \;\text{y}$$

(14) $$\text{cov}({\text{z}}_{\text{y}}-{\text{z}}_{\text{y}-n})=\rho (n),\;\forall \;\text{y}$$

(15) $$\text{var}({\text{z}}_{\text{y}})=\sigma_{\text{z}},\;0<\sigma_{\text{z}}<\infty$$

Given these assumptions, a DM test with equivalent accuracy of forecasts is proposed.

(16) $$\text{DM}=\frac{\overset{¯}{\text{z}}}{{\overset{ˆ}{\sigma }}_{\overset{¯}{\text{z}}}}\overset{n}{\to }\text{Norm}(0,1)$$

In the given formula (16), $\overset{¯}{\text{z}}=\frac{1}{\text{y}}\sum_{\text{y}=1}^{\text{y}}{\text{z}}_{\text{y}}$ is the mean sample loss difference, and ${\overset{ˆ}{\sigma }}_{\overset{¯}{\text{z}}}$ is a consistent standard error estimate of ${\text{z}}_{\text{y}}$.

Ultimately, this study demonstrates the superiority of the suggested hybrid combination forecasting technique using various figures such as the line plot, correlogram plot (autocorrelation and partial autocorrelation functions), bar plot, and dot plot.

Finally, combining the long-run curve trend series forecast and the residual series forecast leads to sixteen possible combinations for comparison purposes $\left(4^{2}=16\right)$. Each combination model with parametric and nonparametric regression models is denoted in this study by ${}^{{\text{L}}_{\text{y}}}$M${}^{{\text{R}}_{\text{y}}}$, where the ${\text{L}}_{\text{y}}$ at the top-left is related with a long run curve trend series, and the ${\text{R}}_{\text{y}}$ represents the residual series at the top-right. This work assigns a code to each model in the forecasting models: “a” for the AR, “b” for the ARMA, “c” for the NPAR, and “d” for the NNA. For example, ^0^M${}^{{\text{a}}_{1}}$ represents the estimate of the long-term trend (${\text{L}}_{\text{y}}$) with the parametric regression (polynomial regression model), and the residual series (${\text{R}}_{\text{y}}$) estimated using the AR model and ^1^M${}^{{\text{a}}_{1}}$ represents the estimate of the long-term trend (${\text{L}}_{\text{y}}$) with the nonparametric regression (smoothing spline regression model), and the residual series (${\text{L}}_{\text{y}}$) estimated using the AR model similarly ^0^M${}^{{\text{b}}_{1}}$ shows parametric regression using ARMA model and ^1^M${}^{{\text{b}}_{1}}$ presents nonparametric model with ARMA and so on ^0^M${}^{{\text{d}}_{1}}$ denotes parametric with neural network auto-regressive and ^1^M${}^{{\text{d}}_{1}}$ represents nonparametric with NNA respectively. Therefore, the different forecasting models are integrated to provide a final year-ahead CO_2_ emission forecast. The final equation is as follows:

(17) $${\overset{ˆ}{\text{C}}}_{\text{y}+1}=\left({\overset{ˆ}{\text{L}}}_{\text{y}+1}+{\overset{ˆ}{\text{R}}}_{\text{y}+1}\right)$$

**Figure 2:**
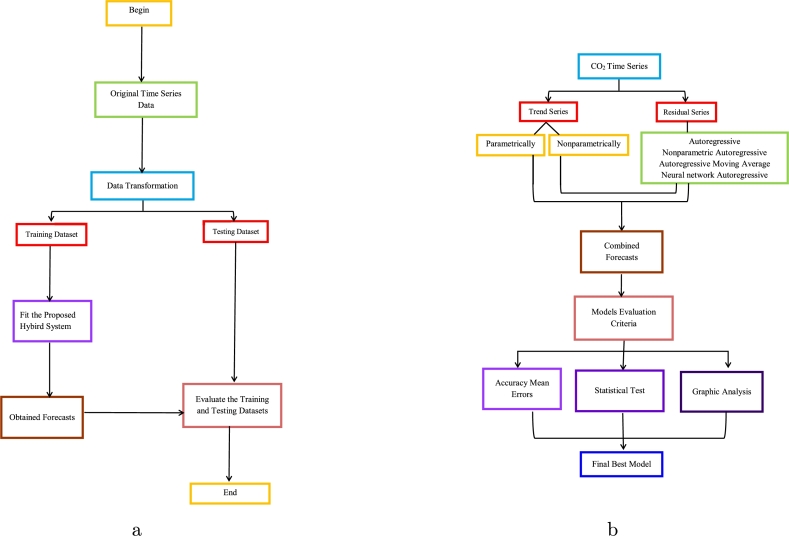
Pakistan Carbon Dioxide Emission (metric tons per capita): (a) the training and testing phases of the proposal.; (b) the proposed hybrid combination modeling and forecasting approach is depicted in a flowchart.

At the end of this section, Fig. 2 depicts the proposed hybrid combination modeling and forecasting strategy to finish this part.

## 3 Case study outcomes

**Figure 3:**
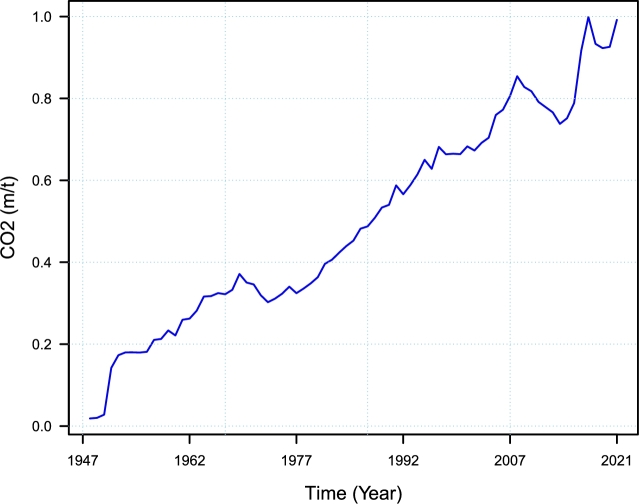
Pakistan Carbon Dioxide Emission (metric tons per capita): The CO_2_ emission time series plot from 1949 to 2021.

This research study compiles CO_2_ emissions in Pakistan from 1949 to 2021. According to international standards, CO_2_ was measured in metric tons per capita, and the dataset was taken from the World Bank website (https://databank.worldbank.org). The dataset's features (descriptive statistics) are shown numerically in Table 1, and the CO_2_ emissions time series is illustrated visually in Fig. 3. According to the descriptive data in Table 1, the mean CO_2_ emissions time series from 1949 to 2021 are 0.4974 metric tons per capita, with a low of 0.0185 metric tons per capita and a maximum of 0.9990 metric tons per capita. On the other hand, the skewness has a 0.1518, and kurtosis has a 0.1997. These values show that the dataset is not normally distributed. In addition, from Fig. 3, it can be observed that there is an increasing upward trend, which indicates a seasonal direction in the time series of CO_2_ emission. In addition, with a gradual increase in CO_2_ emission from 1949 to 1971 and attempted the first peak in 1972. After this, the trend slightly decreased during 1975, and again it took an increasing trend and the 2nd highest peak attempt in 2007. After the second peak, again a downward fall trend for a short period in 2011. Similarly, there was an increasing CO_2_ emission trend and another peak attempt in 2019, while a slightly downward trend was observed in 2020. The region's separation mainly caused these fluctuations, the unhealthy political environment, and unsuccessful plan implementations. Hence, in the proposed hybrid combination forecasting approach, we remove the long-run trend series using the parametric and nonparametric models to achieve this. It is worth noting that the stationarity of time series data is generally checked using the Augmented Dickey-Fuller, Unit root, and Philips-Perron tests. However, some studies have demonstrated that these tests may provide skewed and deceptive findings due to the possibility of structural discontinuities in the time series data [60]. We did not employ these tests since the residual series is practically stationary after filtering the CO_2_ emission series for long run curve trend series.

**Table 1:** Pakistan Carbon Dioxide Emission (metric tons per capita): CO_2_ time series descriptive statistics in Pakistan from 1949 to 2021.

S. No	Statistic	Value
1	Minimum	0.0185
2	Q1	0.3168
3	Median	0.4527
4	Mean	0.4974
5	Q3	0.6978
6	Max	0.999
7	ADF	0.4774 (-2.2405)
8	Skewness	0.1518223
9	Kurtosis	1.977317

**Figure 4:**
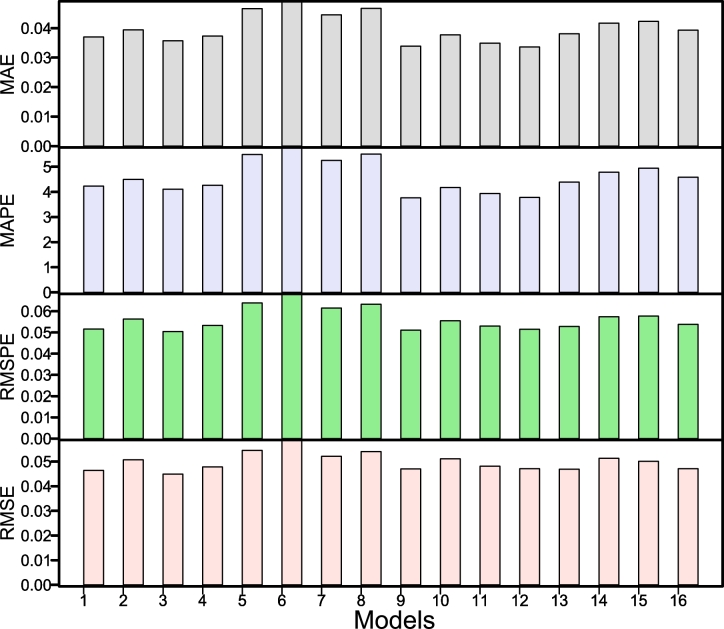
Pakistan Carbon Dioxide Emission (metric tons per capita): The bar plot of the out-of-sample accuracy mean errors of CO_2_ emission forecast for all sixteen hybrid combination models.

**Figure 5:**
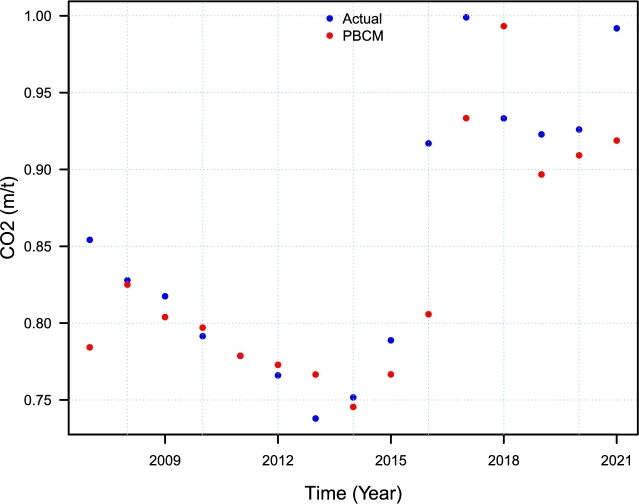
Pakistan Carbon Dioxide Emission (metric tons per capita): Actual and forecasted CO_2_ emission for only the final best model ^1^M${}^{{\text{a}}_{0}}$ for January 2007–December 2021.

To produce a year-ahead forecast for CO_2_ emissions using the proposed hybrid combination forecasting approach outlined in Section 2, the following actions had to be taken: First, the parametric and nonparametric regression models were employed to generate a long-run curve trend series (L_y_) and a residual series (R_y_). Second, four conventional time series models were applied to each subseries: the parametric autoregressive, the autoregressive moving average model, the nonparametric autoregressive model, and the neural network autoregressive model. The model parameters were then determined, and the one-year prediction was derived using the expanding window approach. Thus, Equation (17) produced the final one-year-ahead CO_2_ emission forecasts. Finally, combining the long-run curve trend series forecast and the residual series forecast leads to sixteen possible combinations for comparison purposes $\left(4^{2}=16\right)$. To evaluate and compare the performance of these sixteen hybrid combination models using three criteria: a) accuracy mean errors, b) statistical tests, and c) graphical presentation. The one-year ahead out-of-sample accuracy mean errors (MAE, MAPE, RMSE, RMSPE) for all sixteen hybrid combination models are listed in Table 2. It is confirmed from this table that the best hybrid combination forecasting model within all the sixteen hybrid combination models is ^1^M${}^{{\text{a}}_{0}}$, which obtained 0.0333, 3.7621, 0.0511, and 0.0470 for MAE, MAPE, RMSPE, and RMSE, respectively. It can be observed that the MAE, MAPE, RMSPE, and RMSE are the lowest values compared to all other combinations. In the same way, the model ^1^M${}^{{\text{d}}_{0}}$ produced the second-best result compared to all other used combination models with 0.0336, 3.7798, 0.0515, and 0.0471 for MAE, MAPE, RMSPE, and RMSE, accordingly. However, the ^1^M${}^{{\text{c}}_{0}}$ model and ^0^M${}^{{\text{c}}_{0}}$ model showed the third and fourth best results (MAE= 0.0349, 0.0357; MAPE= 3.9356, 4.1071; RMSPE= 0.0504, 0.0530; RMSE= 0.0481, .0449). Once the best hybrid combination model was obtained using the accuracy mean errors, check their consistency using a statistical test. To do this, the DM test was carried out for each pair of models to confirm the superiority of the findings stated in Table 2. The DM test findings (emphasis p-values) are shown in Table 3. Each item in the table represents the p-value of a hypothesis system. The null hypothesis assumes no difference in the model's accuracy in the column/row vs. the alternative hypothesis that the model in the column is more accurate than the model in the row. This table shows that, among all sixteen hybrid combination models, the ^1^M${}^{{\text{a}}_{0}}$, ^1^M${}^{{\text{d}}_{0}}$, and ^1^M${}^{{\text{c}}_{0}}$ models at a 10% significance level are statically better than the rest, except when comparing them to ^0^M${}^{{\text{c}}_{0}}$. Finally, to check the graphical performance of the best models, as mentioned previously, this work presents the bar plots for the accuracy of mean errors. For instance, in Fig. 4, the MAE, the MAPE, the RMSPE, and the RMSE are plotted. For this figure, can seen that the ^1^M${}^{{\text{a}}_{0}}$, ^1^M${}^{{\text{d}}_{0}}$, ^1^M${}^{{\text{c}}_{0}}$, and ^0^M${}^{{\text{c}}_{0}}$ show minimum accuracy mean errors as compare the rest of all models. In addition, forecasted values from the best hybrid combination model (^1^M${}^{{\text{a}}_{0}}$) among all sixteen combination hybrid models are plotted in Fig. 5. This plot shows that the predicted values follow the observed values of CO_2_ emission very well. On the other hand, Fig. 7 depicts the autocorrelation (ACF) and partial autocorrelation (PACF) plots of the residuals for the final best hybrid combination model (^1^M${}^{{\text{a}}_{0}}$). The series no longer shows a notable autocorrelation structure in both figures, indicating that it has been whitened and is satisfactory. Thus, it is concluded from these findings (mean errors, statistical test, and graphical analysis) that the proposed hybrid combination forecasting technique is highly accurate and efficient in forecasting CO_2_ emissions.

**Table 2:** Pakistan Carbon Dioxide Emission (metric tons per capita): One-year-ahead out-of-sample accuracy mean errors of CO_2_ emission forecast for all sixteen hybrid combination models.

**S.No**	**Models**	**MAE**	**MAPE**	**RMSPE**	**RMSE**
1	^0^M${}^{{\text{a}}_{0}}$	0.0370	4.2284	0.0516	0.0464
2	^0^M${}^{{\text{b}}_{0}}$	0.0394	4.4982	0.0563	0.0507
3	^0^M${}^{{\text{c}}_{0}}$	0.0357	4.1071	0.0504	0.0449
4	^0^M${}^{{\text{d}}_{0}}$	0.0373	4.2621	0.0533	0.0478
5	^0^M${}^{{\text{a}}_{1}}$	0.0466	5.4875	0.0639	0.0545
6	^0^M${}^{{\text{b}}_{1}}$	0.0491	5.7648	0.0682	0.0587
7	^0^M${}^{{\text{c}}_{1}}$	0.0445	5.2540	0.0615	0.0521
8	^0^M${}^{{\text{d}}_{1}}$	0.0467	5.5058	0.0633	0.0540
9	^1^M${}^{{\text{a}}_{0}}$	0.0333	3.7621	0.0511	0.0470
10	^1^M${}^{{\text{b}}_{0}}$	0.0377	4.1753	0.0555	0.0511
11	^1^M${}^{{\text{c}}_{0}}$	0.0349	3.9356	0.0530	0.0481
12	^1^M${}^{{\text{d}}_{0}}$	0.0336	3.7798	0.0515	0.0471
13	^1^M${}^{{\text{a}}_{1}}$	0.0381	4.3899	0.0528	0.0469
14	^1^M${}^{{\text{b}}_{1}}$	0.0417	4.7810	0.0574	0.0513
15	^1^M${}^{{\text{c}}_{1}}$	0.0423	4.9423	0.0577	0.0501
16	^1^M${}^{{\text{d}}_{1}}$	0.0393	4.5840	0.0538	0.0471

**Table 3:** Pakistan Carbon Dioxide Emission (metric tons per capita): Results (p-values) of the DM test for all combination models within the proposed filtering and ensemble technique.

**Models**	^0^M${}^{{\text{a}}_{0}}$	^0^M${}^{{\text{b}}_{0}}$	^0^M${}^{{\text{c}}_{0}}$	^0^M${}^{{\text{d}}_{0}}$	^0^M${}^{{\text{a}}_{1}}$	^0^M${}^{{\text{b}}_{1}}$	^0^M${}^{{\text{c}}_{1}}$	^0^M${}^{{\text{d}}_{1}}$	^1^M${}^{{\text{a}}_{0}}$	^1^M${}^{{\text{b}}_{0}}$	^1^M${}^{{\text{c}}_{0}}$	^1^M${}^{{\text{d}}_{0}}$	^1^M${}^{{\text{a}}_{1}}$	^1^M${}^{{\text{b}}_{1}}$	^1^M${}^{{\text{c}}_{1}}$	^1^M${}^{{\text{d}}_{1}}$
^0^M${}^{{\text{a}}_{0}}$	0	0.8799	0.2161	0.6342	0.7093	0.8895	0.8425	0.5554	0.9552	0.9639	0.8749	0.9363	0.5684	0.9111	0.6384	0.5448
^0^M${}^{{\text{b}}_{0}}$	0.1201	0	0.1375	0.3406	0.1494	0.7486	0.4599	0.3255	0.7823	0.9585	0.5921	0.6845	0.2588	0.5443	0.3647	0.3411
^0^M${}^{{\text{c}}_{0}}$	0.7839	0.8625	0	0.8207	0.8465	0.8757	0.9696	0.7207	0.9637	0.9582	0.9361	0.9841	0.7284	0.9036	0.7784	0.6619
^0^M${}^{{\text{d}}_{0}}$	0.3658	0.6594	0.1793	0	0.4127	0.6825	0.7534	0.3978	0.8216	0.8712	0.7419	0.9	0.4013	0.7224	0.5472	0.387
^0^M${}^{{\text{a}}_{1}}$	0.2907	0.8506	0.1535	0.5873	0	0.874	0.8214	0.5196	0.9695	0.9685	0.893	0.9459	0.5107	0.8506	0.5932	0.5141
^0^M${}^{{\text{b}}_{1}}$	0.1105	0.2514	0.1243	0.3175	0.126	0	0.4241	0.3017	0.7685	0.9714	0.5577	0.6576	0.2475	0.4777	0.3447	0.3239
^0^M${}^{{\text{c}}_{1}}$	0.1575	0.5401	0.0304	0.2466	0.1786	0.5759	0	0.1313	0.7927	0.8564	0.6703	0.8809	0.2569	0.5663	0.3155	0.2783
^0^M${}^{{\text{d}}_{1}}$	0.4446	0.6745	0.2793	0.6022	0.4804	0.6983	0.8687	0	0.8593	0.8831	0.8011	0.9631	0.49	0.708	0.595	0.4987
^1^M${}^{{\text{a}}_{0}}$	0.0448	0.2177	0.0363	0.1784	0.0305	0.2315	0.2073	0.1407	0	0.8763	0.1848	0.4459	0.1564	0.3065	0.2226	0.2104
^1^M${}^{{\text{b}}_{0}}$	0.0361	0.0415	0.0418	0.1288	0.0315	0.0286	0.1436	0.1169	0.1237	0	0.1183	0.2477	0.1043	0.1495	0.1574	0.1569
^1^M${}^{{\text{c}}_{0}}$	0.1251	0.4079	0.0639	0.2581	0.107	0.4423	0.3297	0.1989	0.8152	0.8817	0	0.7272	0.2439	0.444	0.3074	0.2801
^1^M${}^{{\text{d}}_{0}}$	0.0637	0.3155	0.0159	0.1	0.0541	0.3424	0.1191	0.0369	0.5541	0.7523	0.2728	0	0.1467	0.3461	0.1797	0.1556
^1^M${}^{{\text{a}}_{1}}$	0.4316	0.7412	0.2716	0.5987	0.4893	0.7525	0.7431	0.51	0.8436	0.8957	0.7561	0.8533	0	0.8955	0.6571	0.5113
^1^M${}^{{\text{b}}_{1}}$	0.0889	0.4557	0.0964	0.2776	0.1494	0.5223	0.4337	0.292	0.6935	0.8505	0.556	0.6539	0.1045	0	0.2888	0.2769
^1^M${}^{{\text{c}}_{1}}$	0.3616	0.6353	0.2216	0.4528	0.4068	0.6553	0.6845	0.405	0.7774	0.8426	0.6926	0.8203	0.3429	0.7112	0	0.3272
^1^M${}^{{\text{d}}_{1}}$	0.4552	0.6589	0.3381	0.613	0.4859	0.6761	0.7217	0.5013	0.7896	0.8431	0.7199	0.8444	0.4887	0.7231	0.6728	0

## 4 Discussion

**Figure 6:**
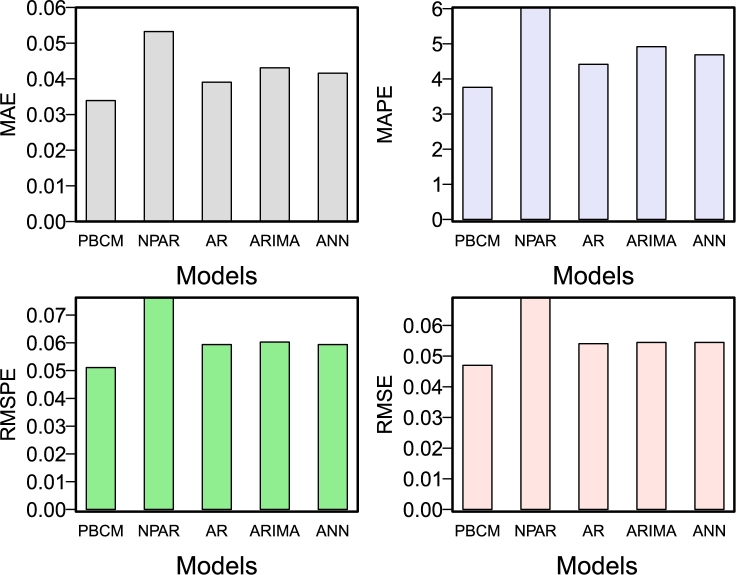
Pakistan CO_2_ emission: The bar plot comparing the proposed best model versus considered benchmark models: Out-of-sample CO_2_ emission accuracy mean forecast errors.

In this research work, the ^1^M${}^{{\text{a}}_{0}}$ model was shown to be the most accurate and efficient for CO_2_ emission forecasting in Pakistan based on the findings of the descriptive statistics, statistical test, and graphical analysis. It is worth mentioning here that the mean accuracy errors (RMSPE, MAPE, MAE, and RMSE) reported in this study were lower than those of the benchmark models, which included the nonparametric autoregressive (NPAR), autoregressive (AR), autoregressive moving integrated average (ARIMA), and neural network autoregression models. Table 4 presents a numerical comparison of the proposed model with the four benchmark models, while Fig. 6 gives a visual comparison. As both presentations prove, this work's proposed optimal combination model (^1^M${}^{{\text{a}}_{0}}$) produced much lower accuracy mean errors than the Benchmark models. For example, the final best hybrid combination model obtained mean accuracy errors MAE = 0.0333, MAPE = 3.7621, RMSPE = 0.0511, and RMSE = 0.0470, all lower than the benchmark models. To summarize, based on all of these findings and the preceding section, the accuracy of the best hybrid combination (^1^M${}^{{\text{a}}_{0}}$) model is relatively high and efficient compared to all sixteen hybrid combinations and all baseline models.

**Table 4:** Pakistan CO_2_ emission Data: Comparison of the proposed best model versus the considered benchmark models: Out-of-sample CO_2_ emission accuracy mean forecast errors.

**S.No**	**Models**	**MAE**	**MAPE**	**RMSPE**	**RMSE**
1	^1^**M**${}^{{\text{a}}_{0}}$	0.0333	3.7621	0.0511	0.0470
2	**NPAR**	0.0533	6.0354	0.0763	0.0691
3	**AR**	0.0391	4.4181	0.0594	0.0540
4	**ARMA**	0.0431	4.9178	0.0603	0.0544
5	**NNA**	0.0415	4.6803	0.0593	0.0544

**Figure 7:**
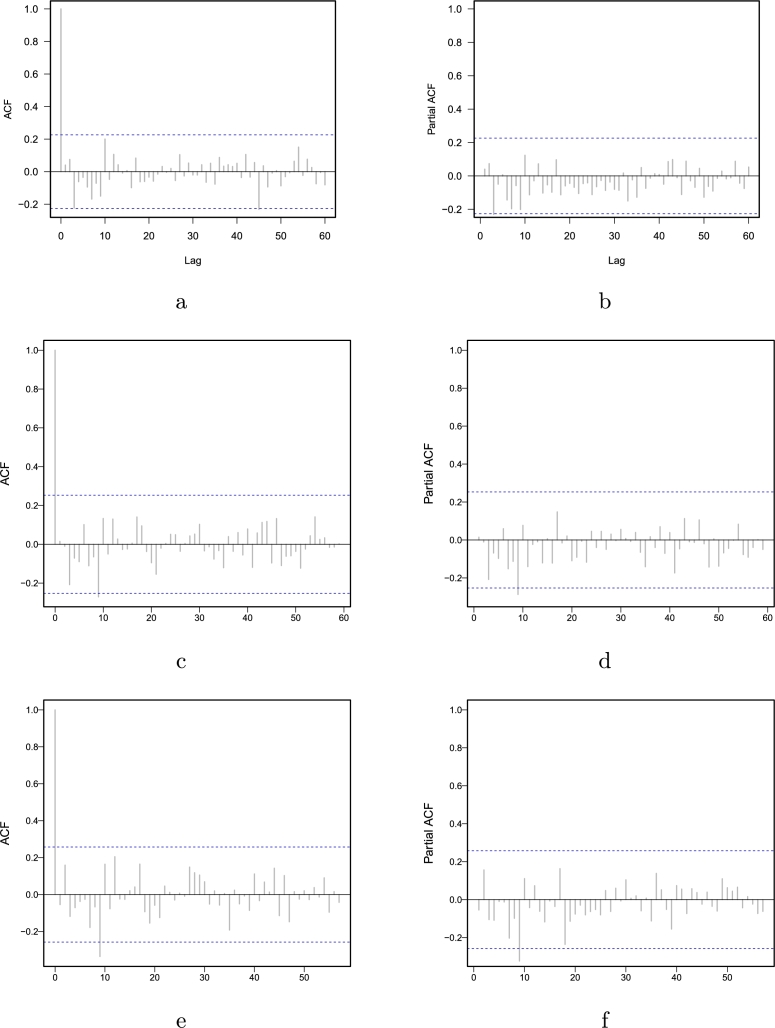
Pakistan Carbon Dioxide Emission (metric tons per capita): The Autocorrelation plot (left), and the partial autocorrelation plot (right) for the three final best hybrid combination models (^1^M${}^{{\text{a}}_{0}}$,^1^M${}^{{\text{d}}_{0}}$, and ^1^M${}^{{\text{c}}_{0}}$), respectively.

Once the best model ^1^M${}^{{\text{a}}_{0}}$ was assessed by descriptive statistics, a statistical test, graphic evaluation, and baseline model comparison, this work proceeded to future forecasting with the superior model. The authors used the ^1^M${}^{{\text{a}}_{0}}$ for the CO_2_ emission in Pakistan and forecast from 2022 to 2030 (using an expanding window forecast). The forecasted CO_2_ emission values are the following: 2022 (1.009671), 2023 (1.024739), 2024 (1.039807), 2025 (1.054875), 2026 (1.069943), 2027 (1.085011), 2028 (1.100079), 2029 (1.115147), 2030 (1.130215), respectively. However, As per UNDP, Pakistan wants to establish a cumulative aggressive conditional objective of a 50% reduction in predicted emissions by 2030, with a 15% reduction coming from domestic resources and a 35% reduction contingent on foreign grant funding. As a result, our findings provide policymakers with valuable information to direct future resource allocation and mitigation efforts. Furthermore, the forecasting exercise will aid in understanding the spread and, as a result, the risk.

In addition, based on the proposed hybrid combination forecasting technique, Pakistan‘s CO_2_ emissions will be 1.130215 metric tonnes per capita by 2030. It implies that the growing emission trend is a worrisome and unmistakable signal that novel strategies to limit this tendency are required. The ever-increasing emission trend is an alarming warning that action must be taken to halt this tendency. To do this, the authors will suggest the Pakistani government adopt the following measures:

• To charge the carbon footprint of enterprises and facilities per tonne and limit the amount of electricity generated from hydroelectricity, wind power, and many other zero-carbon sources.
• To work on population reduction and encourage afforestation in heavily populated areas instead of wooded regions.
• Encourage enterprises, organizations, groups, and people to employ clean technology, create clean and creative zero- or low-carbon technologies, and support research and other research.
• The government of Pakistan lacks sufficient engagement and support on a global platform to address the issue of climate change, and the level of commitment is unclear. Pakistan needs proactive engagement with international climate forums to secure funds and strengthen partnerships with the global community to tackle this global problem.
• Pakistan must engage dedicated and robust institutions to collaborate and initiate coordination efforts among various stakeholders, scientific organizations, civil society, and the private sector to implement, monitor policies, and streamline decision-making in impactful and coherent climate change strategies.
• Pakistan's current policy on climate change focuses only on adaptation measures. It should be expanded to other contributing factors in climate change, including urbanization, unsustainable practices in agriculture sectors, and emissions due to industries. It needs a comprehensive policy on promoting sustainable land use, responsible agricultural practices, urban planning, prioritization of green infrastructure, and strict regulations on industrial emissions.

## 5 Conclusion and future directions

Currently, the CO_2_ emissions continue to rise globally despite efforts to combat climate change. Energy industry emissions are a pressing global issue, causing devastating impacts. Hence, it is vital to accurately and efficiently forecast CO_2_ emissions. Thus, this study has comprehensively analyzed predicting CO_2_ emissions by comparing various hybrid combinations of regression and time series methods to explore the CO_2_ emissions in Pakistan and, first, divided the yearly time series of CO_2_ emissions into the long-run curve trend series and the residual subseries. The long-run curve trend subseries were modeled using parametric and nonparametric regression methods, while various standard time series models were used to forecast the residual subseries. However, the forecasts of each subseries have been combined to obtain the final estimates of CO_2_ emissions. This work used four different accuracy mean errors, a statistical test, and a graphical analysis as performance measures to evaluate the proposed hybrid forecasting technique. The findings confirmed that the proposed hybrid combination forecasting technique is highly accurate and efficient in forecasting CO_2_ emissions. Likewise, according to the proposed final optimal hybrid combination forecasting model, Pakistan's per capita CO_2_ emissions will be 1.130215 metric tons in 2030. Pakistan's escalating emission trend signals that creative solutions must be implemented to curb it. Thus, Pakistan's growing emission pattern signals that creative actions are required to mitigate its impact. The government must tax enterprises’ carbon footprints per ton, regulate zero-carbon power generation, and reduce population. Advocacy efforts will center on afforestation in densely populated regions, clean technology adoption, breakthrough low- or zero-carbon technology development, and research financing.

However, the study's main weakness is that it only incorporates CO_2_ emission data and does not include additional exogenous components such as power pricing, temperature, wind speed, natural gas prices, and so on, which might improve CO_2_ forecasting accuracy. In contrast, the current study employed solely data from Pakistan. Consequently, it may be used in other countries to assess the utility of the suggested hybrid combination modeling and forecasting method. Furthermore, while this study only employed parametric and nonparametric univariate models, machine learning techniques like deep learning and artificial neural networks might be explored within the proposed forecasting framework. On the other hand, it may also be expanded and used for other datasets, such as electrical power systems [61], [62], time-tracking control systems [63], [64], and air pollution [65].

## Funding

This research received no external funding.

## CRediT authorship contribution statement

**Hasnain Iftikhar:** Writing – original draft, Visualization, Validation, Supervision, Software, Resources, Project administration, Methodology, Investigation, Conceptualization. **Murad Khan:** Writing – review & editing, Visualization, Investigation, Formal analysis, Data curation. **Justyna Żywiołek:** Writing – review & editing, Project administration, Investigation, Funding acquisition. **Mehak Khan:** Writing – review & editing, Validation, Investigation. **Javier Linkolk López-Gonzales:** Writing – review & editing, Supervision, Project administration, Resources.

## Declaration of Competing Interest

The authors declare that they have no known competing financial interests or personal relationships that could have appeared to influence the work reported in this paper.

## Data Availability

The data used in this study is openly available on the World Bank website (https://databank.worldbank.org).
